# Development and Validation of SCACOMS, a Composite Scale for Assessing Disease Progression and Treatment Effects in Spinocerebellar Ataxia

**DOI:** 10.1007/s12311-024-01697-8

**Published:** 2024-05-07

**Authors:** Gilbert L’Italien, Evan Popoff, Basia Rogula, Lauren Powell, Michele Potashman, Sam Dickson, Patrick O’Keefe, Melissa Beiner, Vlad Coric, Susan Perlman, Jeremy D. Schmahmann, Suzanne Hendrix

**Affiliations:** 1 Biohaven Pharmaceuticals, Inc 215 Church St, New Haven, CT USA; 2 Broadstreet Health Economics and Outcomes Research, 201-343 Railway Street, Vancouver, BC Canada; 3 Department of Neurology, University of California Los Angeles, Los Angeles, CA USA; 4 Ataxia Center, Laboratory for Neuroanatomy and Cerebellar Neurobiology, Department of Neurology, Massachusetts General Hospital, Boston, MA USA; 5 Pentara Corp, 2261 East 3300 South, Millcreek, UT USA

**Keywords:** Spinocerebellar Ataxia, Partial Least Squares Regression, Composite Measure, Disease Progression

## Abstract

Spinocerebellar ataxias (SCA) are rare inherited neurodegenerative disorders characterized by a progressive impairment of gait, balance, limb coordination, and speech. There is currently no composite scale that includes multiple aspects of the SCA experience to assess disease progression and treatment effects. Applying the method of partial least squares (PLS) regression, we developed the Spinocerebellar Ataxia Composite Scale (SCACOMS) from two SCA natural history datasets (NCT01060371, NCT02440763). PLS regression selected items based on their ability to detect clinical decline, with optimized weights based on the item’s degree of progression. Following model validation, SCACOMS was leveraged to examine disease progression and treatment effects in a 48-week SCA clinical trial cohort (NCT03701399). Items from the Clinical Global Impression—Global Improvement Scale (CGI-I), the Friedreich Ataxia Rating Scale (FARS) – functional stage, and the Modified Functional Scale for the Assessment and Rating of Ataxia (f-SARA) were objectively selected with weightings based on their sensitivity to clinical decline. The resulting SCACOMS exhibited improved sensitivity to disease progression and greater treatment effects (compared to the original scales from which they were derived) in a 48-week clinical trial of a novel therapeutic agent. The trial analyses also provided a SCACOMS-derived estimate of the temporal delay in SCA disease progression. SCACOMS is a useful composite measure, effectively capturing disease progression and highlighting treatment effects in patients with SCA. SCACOMS will be a powerful tool in future studies given its sensitivity to clinical decline and ability to detect a meaningful clinical impact of disease-modifying treatments.

**Supplementary Information:**

The online version contains supplementary material available at 10.1007/s12311-024-01697-8.

## Introduction

Spinocerebellar ataxias (SCAs) constitute a genetically heterogeneous group of autosomal dominant inherited neurodegenerative disorders [1–3]. SCA3 is the most prevalent SCA genotype among almost 50 subtypes categorized genetically into repeat expansions or non-repeat mutations [1–3]. SCAs are progressive diseases in which the cerebellum slowly degenerates; other parts of the nervous system, such as the spinal cord, basal ganglia, and pontine nuclei in the brainstem may be involved as well, depending on the specific subtype [4]. Consequently, patients with SCAs present with imbalance and gait ataxia, incoordination of the limbs, visual disorders, and dysarthria [5]. Other clinical features, including pyramidal and extrapyramidal signs, ophthalmoplegia, and cognitive impairment may also be present. Disease onset occurs typically in the third or fourth decades of life, often culminating in substantial long-term disability [3, 4]. In a longitudinal cohort study, the 10-year survival rate for patients with SCA differed by genotype: 57% (95% CI 47–69) for SCA1, 74% (67–81) for SCA2, 73% (65–82) for SCA3, and 87% (80–94) for SCA6 [6].

There is currently no disease modifying treatment approved for SCA, leaving clinical care of patients focused primarily on symptom management [3, 7]. Advances in understanding the pathophysiological mechanisms underpinning SCA have spurred the development of disease-modifying treatment such as treatments targeting the underlying pathophysiology of SCA and gene therapies [4, 5]. However, many of these treatments have yet to be tested in patients with SCA, and there are numerous challenges in clinical trial development rooted in the low prevalence and genetic heterogeneity of SCAs [8, 9].

Due to the broad range of symptoms and variable disease progression rates among SCA patients, measuring clinically meaningful changes in response to disease-modifying treatments has been difficult, especially without knowledge of which aspects of disease best characterize disease progression at any given stage. Currently, ataxia rating scales, performance tests, functional assessments, and measures of health-related quality of life (HRQoL) are utilized to capture a comprehensive clinical picture over the course of the disease [8]. Existing scales targeting focused symptoms and signs do not capture the complex array of neurodegenerative manifestations and effects, and therefore may fail to detect small yet meaningful changes in clinical status in early-stage disease when progression is slow [10, 11].

A more sensitive measure of clinical decline derived from existing scales would allow for the design of robust studies in SCA and would enable effective disease modifying treatments (DMTs) to reach patients more efficiently. Estimates of delay in disease progression, which is a relevant metric for evaluating DMTs, can also be derived from the composite scale. The purpose of this study was to utilize methodology previously applied in the development of the Alzheimer’s Disease Composite Score (ADCOMS), and analogous to methods used in the development of the composite Unified Huntington Disease Rating Scale (cUHDRS), to introduce a composite clinical rating scale for the SCAs that is objectively optimized to measure disease progression and that provides an estimate of the temporal delay in disease progression with effective treatment.

## Methods

A composite clinical rating scale, the Spinocerebellar Ataxias Composite Score (SCACOMS), was developed based on the approach described by Wang et al. (2016), used to create the ADCOMS in Alzheimer’s disease [12]. The methodology permits the objective selection and weighting of measures based on their responsiveness to disease progression as observed in natural history studies. The resultant composite scale is highly sensitive to disease progression, a key requirement in psychometric scale validation [13]. The approach involves the application of partial least squares (PLS) regression and data from natural history studies to derive the scale. The availability of two natural history data sources allows for cross-validation of the composite scale. This composite scale is then applied to data from a clinical trial assessing an experimental SCA therapy, to estimate the treatment effect and associated temporal delay in disease progression with treatment. The goal was to develop an optimally weighted composite score for measuring disease progression over 48-weeks in mild-to-moderate stage SCA patients.

PLS regression can be considered a construct of two other techniques: principal components regression and reduced rank regression. Principal components regression was developed to allow for correlated variables in regression and identification of a smaller set of uncorrelated variables for applied use. In principal components regression, selected factors are optimized based on predictor variation, while reduced rank regression selects factors optimized to describe response variation. PLS regression balances explanatory predictor variation with explanatory response variation; thus, the correlation between the composite variables and the response variable is greater than with principal components regression, and the method will identify composites with high responsiveness and low bias.

### Data Sources

Two natural history studies—the Clinical Research Consortium for Spinocerebellar Ataxias (CRC-SCA; NCT01060371) and the European Integrated Project on Spinocerebellar Ataxias (EUROSCA; NCT02440763) provided ample data on longitudinal disease progression with multiple years of follow up, and were used to establish the natural progression of SCAs [14–16].

CRC-SCA is a natural history study with the primary objective of describing the clinical characteristics and progression of symptomatic, genetically confirmed, untreated SCA patients, specifically individuals aged 6 years and above with SCA 1, 2, 3, 6, 7, 8, and 10 (Table 1) [14]. A total of 650 participants were recruited across 14 centers in the United States, and longitudinal evaluations were conducted at 12-month intervals. Outcomes collected included ataxia progression as measured by SARA, along with functional assessments, patient-reported outcome measures, and clinical measures. Data were collected from 2010 to the present.

**Table 1:** Overview of natural history studies used to derive SCACOMS

	CRC-SCA	EUROSCA
Sample size		
Baseline	661	423
6 months	232	2
12 months	257	367
18 months	92	13
24 months	87	350
Scales examined*		
Ataxia	SARA^a^	SARA^a^
Activities of daily living	FARS-ADL^b^	–
Functional	FARS-FUNC^c^	–
Clinical	CGI^d^	CGI^d^

^*^To allow for testing of SCACOMS in the clinical trial dataset, scales that were used in both BHV4157-206 and the natural history sets were prioritized for inclusion in the composite endpoint analysis

^a^ Includes items relating to gait, stance, sitting, speech

^b^ Includes items relating to speech, swallow, food, dress, hygiene, falling, walking, sitting

^c^ Six stages of functional status

^d^ Likert scale of global improvement

CGI, Clinical Global Impression—Global Improvement Scale; CRC-SCA, Clinical Research Consortium for the Study of Cerebellar Ataxia; EUROSCA, European Integrated Project on Spinocerebellar Ataxias; FARS-ADL, Friedreich’s ataxia rating scale-activities of daily living; FARS-FUNC, Friedreich ataxia rating scale-function; SARA, Scale for the Assessment and Rating of Ataxia; UHRDS, Unified Huntington's Disease Rating Scale

EUROSCA is a European natural history cohort study initiated in 2005 to understand the natural history of SCA and to identify prognostic factors in patients with SCA genotypes 1, 2, 3, and 6 (Table 1) [15, 16]. EUROSCA comprises clinical and clinical-genetic data pertaining to SCA. Outcomes collected included ataxia progression (SARA), along with other functional and clinical measures. Participants were recruited across 17 centers with evaluations conducted at 12-month intervals. Data on 423 subjects with SCA were available for the current analysis. For both natural history datasets, the follow up period for the PLS regression analyses was from baseline to two years.

The derived and validated SCACOMS were applied to estimate treatment effects among patients enrolled in the BHV4157-206 (NCT03701399) study dataset, a phase 3, multicenter, randomized, double-blind, placebo-controlled parallel-group study designed to assess the safety, tolerability, and efficacy of troriluzole in adults with SCA genotypes 1, 2, 3, 6, 7, 8, and 10 [17]. Subjects were randomized to receive placebo or troriluzole and were stratified by SCA genotype. The primary objective was to compare the efficacy of troriluzole vs placebo on ataxia symptoms in subjects with SCA after 48 weeks of treatment, as measured by the total score on the f-SARA, a modified version of SARA developed for use in the clinical trial setting [17]. Despite minimal change in the primary end point in the overall study population, post-hoc analyses in a pre-specified subgroup of patients with SCA3 showed consistent treatment benefits across multiple outcome measures including the change from baseline f-SARA at Week 48, CGI total score at Week 48, and a robust reduction in fall risk over the study period. In total, SCA3 subjects represented 41% of study participants [18].

### Analyses

Within the CRC-SCA and EUROSCA databases, the population was restricted to align with the inclusion and exclusion criteria of the BHV4157-206 clinical study, specifically, baseline SARA gait of 1 to 7. Analyses included patients with available data for all included model variables at baseline and 12 or 24 months. The resultant analytic sample sizes in the natural history datasets for the derivation of four SCACOMS models were of n = 214 for CRC-SCA all SCA genotypes, n = 77 for CRC-SCA SCA3 genotype only, n = 423 for EURO-SCA all SCA genotypes, and v = 106 for EURO-SCA SCA3 genotype only.

#### Data preparation

Candidate scales deemed sensitive to disease progression and convergent with items included in the BHV4157-206 study were identified within the natural history studies as SARA, Friedreich ataxia rating scales-activities of daily living (FARS-ADL), the Friedreich ataxia rating scale-functional staging (FARS-FUNC) and the CGI. Since the SARA was common in both natural history datasets, and the f-SARA was utilized in BHV-4157–206, the latter was mapped from SARA in the natural history data sets. Specifically, the first four items of SARA (gait, balance, sitting, and speech) were rescaled to mirror the 5-point scaling options of f-SARA. This derivation was similar to the method by Moulaire et al. 2023 [11]. Further details on these scales, and mapping algorithm is available in the *electronic supplementary material*.

In general, each item from the original scales could serve as candidate items in the derivation of the composite, however there were differences in scale availability between CRC-SCA and EUROSCA. For the CRC-SCA dataset, item selection incorporated all items from f-SARA, FARS-FUNC, and CGI. Although FARS-ADL items were of interest, limited data availability prohibited use. For EUROSCA, the selection of items included all items from f-SARA and the CGI, as FARS-FUNC and FARS-ADL were not collected. Other scales were examined to evaluate whether a robust proxy may be included, however these were not available.

Prior to examining item performance in PLS regression models, the item-level scores were standardized across scales by transforming scores into a 0 to 1 scoring range. A score of 0 uniformly reflected the best possible score and a score of 1 reflected the worst possible score. Additional details on data preparation are in the *electronic supplemental material.*

#### PLS regression

To assess disease progression from individual candidate scale items, linear decline models were fit using PLS regression techniques (R v4.2.1) [12, 19, 20]. A linear decline model was deemed a valid representation of disease progression as the population was constrained to patients within the linear phase of an overall sigmoidal decline observed over the follow up period [10].

In deriving composite scales using PLS regression, time is considered the dependent variable as a measure of disease progression during the period of linear decline. Items derived from the regression analysis and their weightings (e.g., f-SARA, FARS-FUNC, CGI) were defined as predictor variables.

Another feature of PLS regression is the application of the variable importance of projection (VIP) and Wold’s criteria to support the selection of relevant candidate items. VIP scores denote a variable's significance in the PLS model and in this analysis, the degree to which that variable influences progression over time in the overall dataset. Wold’s criteria is the threshold used to evaluate the significance of the items. VIP scores are calculated for a given variable as a sum of its squared PLS weights, weighted by the percentage of variance in the outcome variable explained by each latent variable. Variables with VIP scores > 0.5 were included and if variables had VIPs that approximated 0.5, these were included if deemed to be clinically relevant and if they contributed over 5% of the total composite score weight. The PLS regression coefficients were then used as weights that reflected the relative contribution towards progression amongst the items. The resultant composite score (SCACOMS) thus reflects a weighted linear combination of the selected individual scale items. To support interpretation of SCACOMS, the mean to standard deviation ratio (MSDR) was calculated as a measure of responsiveness. MSDRs are defined as the change from baseline (CFB) for each item at follow up divided by the standard deviation for the CFB. MSDRs are essentially identical to Standardized Response Means (SRM) utilized in psychometric scale validation. MSDRs were estimated for both individual items in SCACOMS and for the total scale.

These regressions were repeated across both datasets, on the full population, and on the SCA3 subset of the population to assess whether the item selection and weights differed among these different cohorts. Additional details on the regression models are provided in the *electronic supplemental material.*

#### Cross-validation

Two cross-validation techniques were performed to assess the reliability of SCACOMS: cross-validation by interchanging weights between CRC-SCA and EURO-SCA and a fivefold split sample analysis within each individual dataset.

Cross-validation by interchanging weights amongst scales involved comparing the MSDR of SCACOMS total scores between the original CRC-SCA and EUROSCA models and after swapping the item weights between datasets (i.e., using CRC-SCA data with EUROSCA PLS regression weights and vice versa). The purpose of cross validation is to discover how much the performance of the scale is expected to decrease when applied in a dataset external to the training set, with some degradation expected.

Since functional stage (FARS-FUNC) was not available in EUROSCA, modifications to this approach were employed: 1) we discounted functional stage data and used the weights for all other variables; and 2) we used the functional stage weight from the CRC-SCA model and redistributed the EUROSCA weights such that functional stage contributed the same original percentage.

A fivefold cross validation was performed by randomly assigning subjects in the natural history datasets into 5 cohorts. Each cohort was systematically omitted, such that the remaining 80% of the pooled study population was used to derive the model (training set). This model was then tested on the remaining 20% (test set). This process was repeated 40 times, with the composition of the training and test sets being randomly allocated in each iteration (40 iterations × fivefold, for 200 estimates total). MSDR values were compared between the training set and the test set within each fold, for each iteration, to estimate the average residual bias of the MSDR in the total population.

#### Evaluation of Treatment effects in BHV4157-206

The following four SCACOMS models were developed and validated:

- CRC-SCA-derived model in all SCA patients applied to SCA3 subgroup of BHV-4157–206
- EUROSCA-derived model in all SCA patients applied to SCA3 subgroup of BHV-4157–206
- CRC-SCA-derived model in SCA3 patients applied to SCA3 subgroup of BHV-4157–206
- EUROSCA-derived model in SCA3 patients applied to SCA3 subgroup of BHV-4157–206

For each of the four models, treatment effects were estimated in the SCA3 subgroup of patients enrolled in BHV4157-206. Since these analyses were *post-hoc* and designed to provide an illustration of the treatment effect, patients were analyzed according to their treatment allocation. Using mixed model repeated measures (MMRM) with fixed effect covariates for treatment, analysis visit, cohort by treatment, and baseline SCACOMS score as covariates, the study evaluated the SCA3 population via SCACOMS at baseline, week 8, week 24 and week 48. In addition, the percent progression avoidance, Cohen’s d, and the estimated delay (months) in progression with troriluzole treatment were calculated [21].

Lastly, sample sizes required to demonstrate a 30% and 50% delay in disease progression, at 80% and 90% power were calculated. Percent delay in disease progression with treatment is used in conjunction with the placebo progression to determine the Cohen’s d for each model. The sample size calculations are based on the effect size and power for a potential SCA clinical trial, when using SCACOMS compared to existing clinical trial scales (e.g., f-SARA, CGI, FARS-FUNC).

## Results

The baseline characteristics of participants in the CRC-SCA and EUROSCA analytic datasets are summarized in Table 2.

**Table 2:** Descriptive data for the CRC-SCA analytic dataset (n = 214) and EUROSCA analytic dataset (n = 423) used to derive SCACOMS

Baseline characteristic	CRC-SCA analytic dataset (n = 214)*	EUROSCA analytic dataset (n = 423)*
Age, mean (SD) years	52.3 (13.4)	47.3 (12.7)
Sex, % female	54.2	53.9
Genotype, %		
SCA1	18.2	23.9
SCA2	20.1	31.9
SCA3	35.5	25.1
SCA6	22.4	19.1
SCA8	2.3	0
SCA10	0.9	0
SCA3 and SCA8	0.5	0
Baseline f-SARA gait score, mean (SD)	1.5 (1.1)	1.6 (1.2)
Baseline f-SARA stance score, mean (SD)	1.3 (1.1)	1.5 (1.3)
Baseline f-SARA speech score, mean (SD)	0.7 (0.9)	1.1 (1.0)
Baseline f-SARA sitting score, mean (SD)	0.5 (0.8)	0.8 (1.0)
Baseline FARS functional stage, mean (SD)	2.8 (1.0)	Not available
Baseline CGI, mean (SD)	NA	NA

^*^Subjects in the analytic dataset were required to have baseline and either 12 or 24 months values on the measures of interest

CGI, Clinical Global Impression—Global Improvement Scale; CRC-SCA, Clinical Research Consortium for the Study of Cerebellar Ataxia; EUROSCA, European Integrated Project on Spinocerebellar Ataxias; FARS, Friedreich ataxia rating scale; f-SARA, Modified functional Scale for the Assessment and Rating of Ataxia; NA, not available; SCA, spinocerebellar ataxia; SD, standard deviation

### Models Derived from All SCA Genotype Patients in Two Natural History Datasets

All derived MSDRs, VIP scores, PLS coefficients (weights), and percent contributions to the total composite score for the models fitted to the CRC-SCA natural history dataset are shown in Table 3 for the all-SCA genotype natural history population. All items except for f-SARA sitting item met the Wold’s VIP cutoff and were retained in the model (Table 3). Among these items, CGI demonstrated the highest percent PLS regression weight, followed by FARS functional stage and the three remaining f-SARA item scores (gait, stance, speech), noting that the distribution of the three f-SARA items were nearly equal in this model. Each item's distribution across relative weightings contributed substantively to the composite score. The SCACOMS score’s overall responsiveness, expressed as MSDR (0.8276), exceeded that of the original f-SARA (MSDR = 0.4826), FARS functional stage (MSDR = 0.3902), and slightly exceeded CGI alone (MSDR = 0.7713).

**Table 3:** VIP scores and PLS coefficients for all SCA patients in the CRC-SCA dataset (n = 214)

Item	MSDR*	VIP	PLS weight	% contribution
f-SARA gait score	0.3975	0.8044	5.0057	14.83
FARS functional stage	0.3902	0.5540	8.7293	25.86
f-SARA stance score	0.2481	1.0414	4.7659	14.12
f-SARA speech score	0.2835	0.6300	3.8264	11.34
f-SARA sitting score	0.1836	0.4652	0.0000	–
CGI	0.7713	1.8298	11.4228	33.85
Overall MSDR	**0.8276**			

CGI, Clinical Global Impression—Global Improvement Scale; CRC-SCA, Clinical Research Consortium for the Study of Cerebellar Ataxia; FARS, Friedreich ataxia rating scale; f-SARA, Modified functional Scale for the Assessment and Rating of Ataxia; MSDR, mean to standard deviation ratio; PLS, partial least squares; SCA, spinocerebellar ataxia; VIP, Variable Importance of Projection

^*^The MSDR of changes in total f-SARA score was 0.4826

In the EUROSCA model for all SCA patients, all items included within the PLS regression model were retained, noting that FARS functional stage was not available (Table 4). CGI contributed the highest weighting, and each of the four f-SARA items contributed substantively to the composite score. The SCACOMS score’s overall responsiveness was high (MSDR = 1.1206), exceeding that of the original f-SARA (MSDR = 0.5117) and slightly exceeding that of CGI (MSDR = 1.1121).

**Table 4:** VIP scores and PLS coefficients for all SCA patients in the EUROSCA dataset (N = 423)

Item	MSDR*	VIP	PLS weight	% contribution
f-SARA gait score	0.3244	0.7828	6.1773	12.26
FARS functional stage	–	–	–	–
f-SARA stance score	0.3791	0.8956	8.3214	16.51
f-SARA speech score	0.2846	0.6321	5.2110	10.34
f-SARA sitting score	0.2587	0.6122	3.9369	7.81
CGI	1.1121	1.6765	26.7503	53.08
Overall MSDR	**1.1206**			

CGI, Clinical Global Impression—Global Improvement Scale; EUROSCA, European Integrated Project on Spinocerebellar Ataxias; FARS, Friedreich ataxia rating scale; f-SARA, Modified functional Scale for the Assessment and Rating of Ataxia; MSDR, mean to standard deviation ratio; PLS, partial least squares; SCA, spinocerebellar ataxia; VIP, Variable Importance of Projection

^*^ The MSDR of changes in total f-SARA score was 0.5117

### Models Derived from SCA3 Patients in Two Natural History Datasets

This process was repeated on the SCA3 patient subset to assess the sensitivity of SCACOMS in this population, across each dataset.

In the CRC-SCA model with SCA3 patients alone, f-SARA sitting exhibited a negative VIP score and was removed (Table 5). Both the FARS functional stage and f-SARA speech scores were slightly below the VIP cutoff of 0.5, therefore models with and without the inclusion of these items were examined. Both were ultimately retained in the final model as they each contributed to > 5% of the final composite score when included and were considered highly relevant from a clinical perspective. The overall MSDR with and without these items was 0.9171 and 0.9327, respectively, resulting in a slight reduction to the overall MSDR (Table 5). In the final composite score, CGI exhibited the highest percent PLS regression weight, followed by FARS functional stage and f-SARA stance score. The SCACOMS responsiveness (0.9171) exceeded that of the original f-SARA (MSDR = 0.5582) and FARS functional stage (0.1688), and slightly exceeded that of the CGI score (0.9125; Table 5).

**Table 5:** VIP scores and PLS coefficients for SCA3 patients in the CRC-SCA dataset (N = 77)

Item	MSDR*	VIP	PLS weight	% contribution
f-SARA gait score	0.4604	0.6417	4.5390	10.45
FARS functional stage	0.1688	0.4741	10.8056	24.87
f-SARA stance score	0.2683	1.0522	9.9743	22.95
f-SARA speech score	0.2614	0.4952	4.2284	9.73
f-SARA sitting score	0.2538	Removed negative coefficient	0.0000	–
CGI	0.9125	1.7353	13.9060	32.00
Overall MSDR	**0.9171**			

CGI, Clinical Global Impression—Global Improvement Scale; CRC-SCA, Clinical Research Consortium for the Study of Cerebellar Ataxia; FARS, Friedreich ataxia rating scale; f-SARA, Modified functional Scale for the Assessment and Rating of Ataxia; MSDR, mean to standard deviation ratio; PLS, partial least squares; SCA, spinocerebellar ataxia; VIP, Variable Importance of Projection

^*^ The MSDR of changes in total f-SARA score was 0.5582

These findings were corroborated in the EUROSCA model, where all items included in the PLS regression model met the Wold’s VIP cutoff of 0.5 (Table 6). CGI contributed the most to the composite score, followed by f-SARA stance and speech. In contrast to the CRC-SCA model, the relative weighting of f-SARA speech and stance were similar. As in the CRC-SCA model, the overall responsiveness of SCACOMS derived from the EUROSCA model for SCA3 patients was high (1.1157), far exceeding that of the original f-SARA (MSDR = 0.5893) and slightly exceeding that of CGI (1.0742).

**Table 6:** VIP scores and PLS coefficients for SCA3 patients in the EUROSCA dataset (N = 106)

Item	MSDR*	VIP	PLS weight	% contribution
f-SARA gait score	0.2952	0.6718	2.5315	4.83
FARS functional stage	–	–	–	–
f-SARA stance score	0.5629	1.0763	13.5141	25.76
f-SARA speech score	0.2320	0.6486	10.7757	20.54
f-SARA sitting score	0.3056	0.5132	2.0793	3.96
CGI	1.0742	1.645	23.562	44.91
Overall MSDR	**1.1157**			

CGI, Clinical Global Impression—Global Improvement Scale; EUROSCA, European Integrated Project on Spinocerebellar Ataxias; FARS, Friedreich ataxia rating scale; f-SARA, Modified functional Scale for the Assessment and Rating of Ataxia; MSDR, mean to standard deviation ratio; PLS, partial least squares; SCA, spinocerebellar ataxia; VIP, Variable Importance of Projection

^*^ The MSDR of changes in total f-SARA score was 0.5893

### Cross-validation of Natural History SCACOMS Composite Scores

Cross-validation of SCACOMS composition across the two natural history datasets yielded comparable overall MSDRs, providing further evidence of the validity of both models (Table 7). Results of the fivefold cross validation are presented in Table 8. Average percent bias ranged from -3.40 to 0.28.

**Table 7:** Cross-validation by interchanging weights of Natural History SCACOMS Composite Scores

Model data source	Original composite score MSDR^a^	Cross-validated composite score MSDR^b^
SCA3 patients in CRC-SCA	0.9327	0.9459 (Remove functional stage)
		0.9345 (Retain functional stage)
SCA3 patients in EUROSCA	1.1157	1.092
All SCA patients in CRC-SC	0.8276	0.8141 (Remove functional stage)
		0.8348 (Retain functional stage)
All SCA patients in EUROSCA	1.1206	1.067

CRC-SCA, Clinical Research Consortium for the Study of Cerebellar Ataxia; EUROSCA, European Integrated Project on Spinocerebellar Ataxias; MSDR, mean to standard deviation ratio; SCA, spinocerebellar ataxia; SCACOMS, Spinocerebellar Ataxia Composite Scale

^a^ MSDR calculated in same natural history population or subpopulation from which it was derived

^b^ MSDR calculated for each model in the alternate natural history population

**Table 8:** Results of fivefold cross validation of SCACOMS composite scores

Model	n	Average percent bias
CRC-SCA ALL SCA	214	-0.95462
CRC-SCA SCA 3	77	-3.39901
EUROSCA ALL SCA	423	0.276139
EUROSCA SCA 3	106	0.125194

CRC-SCA, Clinical Research Consortium for the Study of Cerebellar Ataxia; EUROSCA, European Integrated Project on Spinocerebellar Ataxias; SCA, spinocerebellar ataxia; SCACOMS, Spinocerebellar Ataxia Composite Scale

### SCACOMS Treatment Effect Estimation in BHV4157-206

**Fig. 1:**
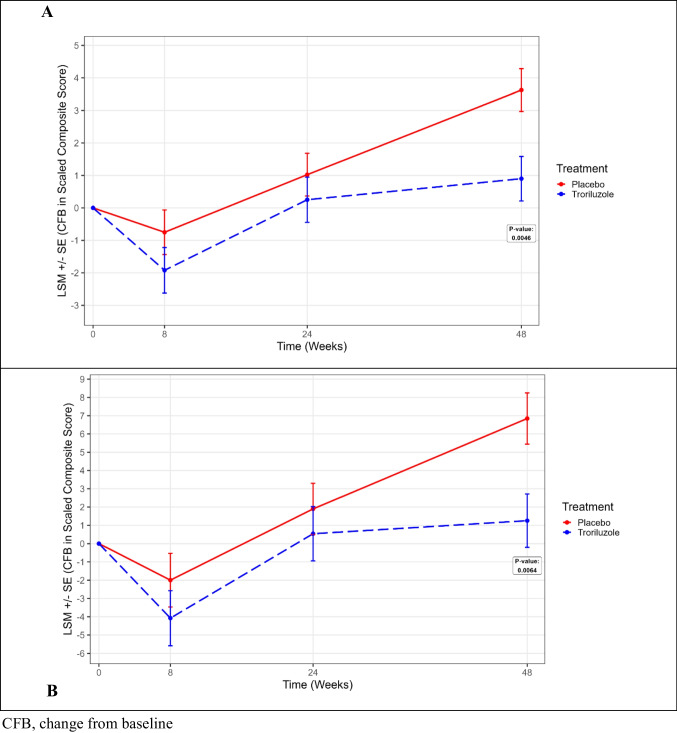
SCACOMS outcomes in BHV4157-206 SCA3 Subjects using (A) CRC-SCA Model and (B) EUROSCA Model derived in All SCA Patients

When applying SCACOMS derived from all SCA patients to BHV4157-206 SCA3 study subjects, a statistically significant difference was observed at 48-weeks with each model derived from the two natural history datasets (Fig. 1). In the CRC-SCA model, the LSM difference for placebo vs troriluzole was 2.73 (SE 0.95; p = 0.0046) (Table 9). In the EUROSCA model, the LSM difference was 5.59 (SE 2.03; p = 0.0064). Moreover, troriluzole was associated with 75% to 82% reduction in disease progression, corresponding to a 6.5- to 7-months delay in progression compared to placebo. Cohen’s d statistics of 0.55–0.56 for the treatment effect provided further evidence of the treatment effect.

**Table 9:** SCACOMS Least Squared Mean Results at 48 weeks for BHV4157-206 SCA3 Subjects Using Model Derived from All SCA Patients in Natural History Datasets

Treatment	LSM	SE	95% CI (lower)	95% CI (upper)	p value
CRC-SCA (all SCA patients) model					
Placebo	3.6254	0.6573	2.3275	4.9234	-
Troriluzole	0.9001	0.6847	-0.4519	2.2521	-
Difference	2.7254	0.9496	0.8503	4.6004	0.0046
Cohen’s d; progression avoided	0.56; 75% of disease progression avoided with troriluzole^a^				
EUROSCA (all SCA patients) model					
Placebo	6.8458	1.4025	4.0777	9.6139	-
Troriluzole	1.2550	1.4611	-1.6287	4.1387	-
Difference	5.5908	2.0274	1.5893	9.5923	0.0064
Cohen’s d; progression avoided	0.55; 82% of disease progression avoided with troriluzole^a^				

^a^ Defined as LSM difference (PBL-TROR)/LSM (PLB) × 100

CI, confidence interval; CRC-SCA, Clinical Research Consortium for the Study of Cerebellar Ataxia; EUROSCA, European Integrated Project on Spinocerebellar Ataxias; LSM, least squared mean; SCA, spinocerebellar ataxia; SCACOMS, Spinocerebellar Ataxia Composite Scale; SE, standard error

**Fig. 2:**
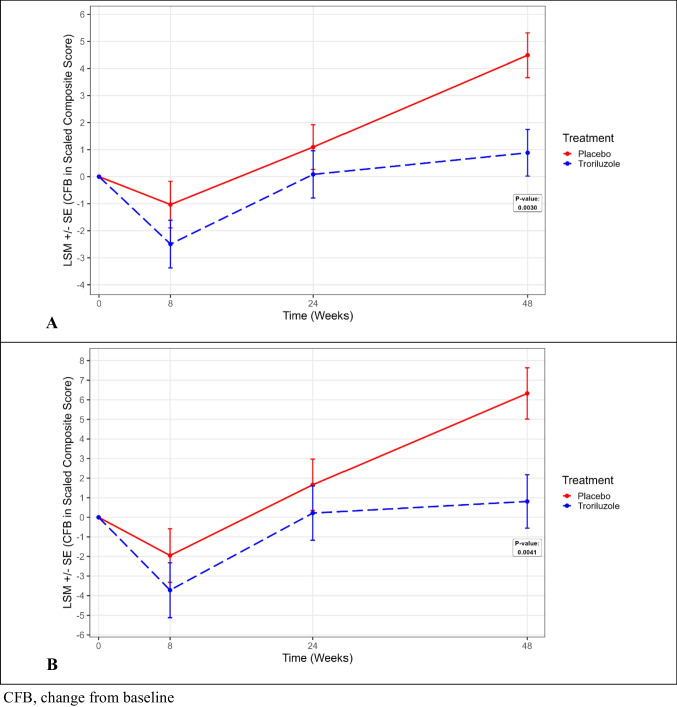
SCACOMS outcomes in BHV4157-206 SCA3 Subjects using (A) CRC-SCA Model and (B) EUROSCA Model derived in SCA3 Patients

Similar findings were observed when applying SCACOMS derived from SCA3 patients to the BHV4157-206 SCA3 study subjects (Fig. 2). In the CRC-SCA model, the LSM difference for placebo vs troriluzole was 3.61 (SE 1.19; p = 0.0030) (Table 10). In the EUROSCA model, the LSM difference was 5.52 (SE 1.90; p = 0.0041). With troriluzole, disease progression avoidance ranged from 80 to 88%, translating to a 7-month delay in progression after one year. Cohen’s d statistics of 0.59 once again underscored the compelling evidence of the treatment effect.

**Table 10:** SCACOMS Least Squared Mean Results at 48 weeks for BHV 4157-206 SCA3 Subjects Using Model Derived from SCA3 Patients in Natural History Datasets

Treatment	LSM	SE	95% CI (lower)	95% CI (upper)	p value
CRC-SCA (SCA3 patients) model					
Placebo	4.4884	0.8262	2.8570	6.1198	-
Troriluzole	0.8833	0.8605	-0.8158	2.5825	-
Difference	3.6051	1.1944	1.2465	5.9637	0.0030
Cohen’s d	0.59; 80% of disease progression avoided with troriluzole^a^				
EUROSCA (SCA3 patients) model					
Placebo	6.3255	1.3113	3.7368	8.9142	-
Troriluzole	0.8086	1.3648	-1.8857	3.5028	-
Difference	5.5169	1.8954	1.7752	9.2586	0.0041
Cohen’s d	0.59; 87% of disease progression avoided with troriluzole^a^				

^a^ Defined as LSM difference (PBL-TROR)/LSM (PLB) × 100

CI, confidence interval; CRC-SCA, Clinical Research Consortium for the Study of Cerebellar Ataxia; EUROSCA, European Integrated Project on Spinocerebellar Ataxias; LSM, least squared mean; SCA, spinocerebellar ataxia; SCACOMS, Spinocerebellar Ataxia Composite Scale; SE, standard error

### Sample Size Calculation

In Table 11 the sample size results are presented. To summarize, SCACOMS had nearly 100% power when utilizing sample sizes that would provide 80% power or higher for the f-SARA, and required sample sizes that were one third the sample size needed for f-SARA for comparable power.

**Table 11:** Sample size required to detect effects for SCACOMS vs f-SARA across different 80% and 90% power, for different genetic groups and natural history samples

Genetic Group	Power	Slowing	Sample Size Required	
			f-SARA	SCACOMS
CRC-SCA sample				
All	80%	0.3	750	256
All	80%	0.5	271	93
All	90%	0.3	1004	342
All	90%	0.5	362	124
SCA3	80%	0.3	561	209
SCA3	80%	0.5	203	76
SCA3	90%	0.3	751	279
SCA3	90%	0.5	271	101
EUROSCA sample				
All	80%	0.3	668	140
All	80%	0.5	241	51
All	90%	0.3	893	187
All	90%	0.5	323	68
SCA3	80%	0.3	504	142
SCA3	80%	0.5	182	52
SCA3	90%	0.3	674	189
SCA3	90%	0.5	244	69

CRC-SCA, Clinical Research Consortium for the Study of Cerebellar Ataxia; EUROSCA, European Integrated Project on Spinocerebellar Ataxias; f-SARA, Modified functional Scale for the Assessment and Rating of Ataxia; SCA, spinocerebellar ataxia; SCACOMS, Spinocerebellar Ataxia Composite Scale; SE, standard error

## Discussion

We used established methodology to develop and validate a new composite scale for SCA, the SCACOMS [12, 22]. The approach utilized PLS regression methods to derive linear longitudinal models that capture disease progression by assessing existing individual scale items within two natural history data sources. The final fitted PLS models included the most responsive scale items using the corresponding PLS coefficients as weighting factors. SCACOMS was applied to clinical trial data in BHV4157-206, which allowed for the estimation of the treatment effects in patients with the SCA3 genotype. The ability of PLS models to select and weight the most highly responsive items is of particular importance in rare diseases such as SCA, in which variable phenotypical presentations and heterogeneity in clinical decline is observed across patients.

The PLS regression models applied to all SCA patients in the CRC-SCA and EUROSCA natural history datasets produced important insights. Directionally consistent findings were observed across the four SCACOMS models developed (in each of the two natural history sets, all SCA genotypes and SCA3 only). CGI accounted for approximately one-third to one-half of the weights across each composite score. Given the potential for heterogeneity of symptoms and signs in patients with SCA, the availability of a ‘global’ item such as the CGI is considered useful for capturing changes that may be unspecified in itemized scales, such as the f-SARA or the FARS functional stage. The f-SARA contributed one-third to one-half of the weights, with the remainder attributable to FARS functional stage in CRC-SCA derived models.

Overall, the total score MSDRs indicated that all SCACOMS endpoints were highly responsive, and validity was confirmed through cross-validation of the two natural history datasets. Although there were some differences in disease progression between the two natural history data sources, this has not exerted a sizeable impact on item selection and weighting, as demonstrated in the cross-validation by interchanging scale weights. The fivefold cross validation results indicate that very little reduction in performance would be expected when SCACOMS is applied in a similar population. Further, when SCACOMS was applied to BHV4157-206 to derive treatment effects for troriluzole vs placebo, the effect size was comparable regardless of which natural history data source SCACOMS was derived from (Cohen’s d: 0.55–0.59).

Application of SCACOMS to the BHV4157-206 SCA3 study subpopulation yielded compelling and consistent treatment effects across all four composite score models at 48-weeks, with a slightly larger treatment effect observed for the SCA3 only SCACOMS. These variations did not impact the overall interpretation of the treatment effect.

Given that clinical trial design in rare progressive diseases can be challenging, the value of a clinically relevant and highly responsive scale cannot be overstated, as this may facilitate the demonstration of a compelling treatment effect for disease modifying therapies, particularly for diseases with a high unmet need. An optimized measure such as SCACOMS can increase statistical power and reduce the sample size and perhaps follow-up time required in an SCA trial, thereby accelerating access to novel therapies for individuals affected with SCA and reducing patient burden in a rare disease. Clinical and patient relevance of the candidate scales have previously been established, strengthening the validity of the re-weighted composite score, and ensuring that SCACOMS items contribute to how a patient “feels/functions/and survives” [23].

Van Dyck et al. adapted the same methodology to estimate the effects of lecanumab in the Phase II/III and Phase III interventional trials in early AD [24]. In those applications, a composite score, ADCOMS, was developed from the most responsive items in a series of cognitive and functional scales used in natural history databases and randomized controlled studies. Similar to Van Dyck et al., the underlying assumption of estimating treatment effects using SCACOMS was that items and scales optimally responsive to natural disease progression would also be responsive to treatments that alter disease progression [24]. This assumption is supported by our findings in both the regression modeling and the generation of treatment effects.

As background, the cUHDRS is widely accepted by the movement disorders community, and was developed using similar methods to the current analysis, using 36 months of data from a longitudinal observational study [25]. In consideration of UHDRS domains, the development of the cUHDRS involved the assessment of signal to noise ratio (which parallels the current MSDR calculations), followed by Pearson correlation and principal components analysis, to define the linear combination of standardized individual variables [25]. This resulted in a scale with enhanced sensitivity to clinical change in early symptomatic disease. PLS regression, used in the current analysis, has its basis in principal components analysis but PLS identifies and selects composite variables based in part on the degree of temporal change, or responsiveness. Therefore, with PLS regression, the associations between the explanatory variables and the dependent variable (temporal change), is enhanced.

Strengths of the present investigation include the use of existing PLS regression methodology and patient-level data from two independent natural history data sources for SCA. Given that SCAs are rare the use of data from this patient population illustrates the validity of our results and enables greater understanding of disease progression in patients with SCA. The estimations of treatment effects were consistent across the four SCACOMS models, regardless of the different natural history sources or differing SCA genotypes. Furthermore, SCACOMS provided an estimate of the delay in disease progression (7 months) associated with treatment until 48 weeks, which is an important metric for evaluating DMTs. Similar findings were demonstrated in the lecanumab trials, where a 6-month delay in cognitive and functional decline was described for AD patients at 18 months of follow up [24].

Certain limitations of this study warrant consideration. A principal goal of developing SCACOMS was to use it to investigate differences between the treatment arms in NCT03701399, which necessitated the use of an algorithm to map a modified SARA score from axial items (gait, stance, sitting, and speech) due to the absence of directly assessed f-SARA scores in the natural history datasets. This poses several challenges. Firstly, the f-SARA was developed as a prospective scale, but the SARA data was mapped to f-SARA data retrospectively. Second, when compared to the SARA, the f-SARA contains fewer items and point categories. Thus, deriving the composite scales using the f-SARA rather than the SARA may have decreased the statistical properties of the composite scale. Third, the f-SARA omits appendicular functions, which may have impacted the clinical importance of the scales, considering that patient experience suggests an impact of impaired appendicular function on quality of life [26]. To explore this limitation further, future work could replicate the analysis using SARA instead of f-SARA, to ascertain differences in item weightings when appendicular items are included. However, such a scale could not be directly applied to BHV4157-206 clinical trial data that utilized f-SARA. Despite these challenges, it is reassuring that a similar mapping of SARA to f-SARA was developed by Moulaire et al. (2023), which lends validity to this method [11].

The candidate scales and items that were used to derive SCACOMS were limited to those assessed in the two natural history databases; however, these do align with measures utilized in clinical trials of SCA. Although FARS functional stage was unavailable in EUROSCA, the cross validation with and without the inclusion of FARS-FUNC in the CRC-SCA models yielded very consistent MSDRs. Additionally, there is potential for information bias associated with the CGI score. This scale is partially subjective as it is based on the overall knowledge of the patient’s condition and abilities, yet potentiallyinfluenced by the investigator’s knowledge on the progressive character of the SCAs. Finally, the derived composite scales are designed to detect maximum differences between two timepoints. While this was effective for the NCT03701399 data, the functional relevance of these changes to patients needs to be explored.

This study highlights the potential usefulness of SCACOMS in both clinical research and practice. It offers a comprehensive assessment of disease progression and treatment effects, and addresses the challenges posed by the heterogeneity of SCA. The greater sensitivity of SCACOMS compared to individual clinical scales underscores its utility in optimizing sample size for clinical trials, thus providing a valuable tool for decision-making and treatment evaluation. Regulatory acceptance and the endorsement by diverse stakeholders, who are motivated to pursue external psychometric and qualitative scale validation, should enhance the validity of SCACOMS and drive its utilization in both trials and natural history data sources.

## Conclusions

We have successfully developed and validated SCACOMS, a new composite clinical rating scale for use in patients with SCA derived from the f-SARA, CGI, FARS-FUNC and FARS-ADL. SCACOMS detects ataxia-related functional impairments in patients with SCA, and most critically, it accurately captures temporal changes in disease progression. Thus, this scale provides an outcome measure that can be used to detect meaningful treatment effects in interventional trials. SCACOMS therefore holds promise for advancing both the understanding and management of these complex and debilitating neurological disorders.

## Supplementary Information

Below is the link to the electronic supplementary material.

Supplementary file1 (DOCX 26 KB)

## Data Availability

To preserve participants privacy, raw data for the natural history and troriluzole datasets are not publicly available. Researchers can submit requests for the CRC-SCA data at https://www.ataxia.org/crc-sca/academic-research/ and EUROSCA data at https://www.eurosca.org.
