# Supplementary material for: Development and Validation of SCACOMS, a Composite Scale for Assessing Disease Progression and Treatment Effects in Spinocerebellar Ataxia
Source: Cerebellum. 2024 May 7;23(5):2028–41. doi: 10.1007/s12311-024-01697-8 (PMC11489241; doi:10.1007/s12311-024-01697-8)
Supplement: Supplementary file 1 — Supplementary file1 (DOCX 26 KB) [file 12311_2024_1697_MOESM1_ESM.docx]

# **Electronic supplementary material**

## The modified Functional Scale for the Assessment and Rating of Ataxia (f-SARA)

| **Score** | **Description** |
| --- | --- |
| **Gait** | |
| *Description:* Proband is asked to walk 10 meters at a safe distance parallel to a wall including a half-turn (turn around to face the opposite direction of gait). If able to walk independently proband is asked to walk 10 steps in tandem (heel to toes) without support. | |
| 0 | Normal: no impairment in walking, turning, and tandem walk |
| 1 | Mildly impaired: abnormal gait, but no assistance needed with walking and/or fails tandem walk |
| 2 | Moderately impaired: staggers, but walks independently (intermittently touches wall, uses examiner’s arm, or uses cane) |
| 3 | Severely impaired: subject dependent upon assistance (requires constant assistance from an accompanying person or uses walker) |
| 4 | Unable to walk, even supported |
| Stance | |
| Description: Item must be performed without shoes. • Utilize the supplied countdown timer. Set the countdown timer to 10 seconds prior to initiating task. [Do not use watch or stopwatch] • Patient is provided up to 3 attempts to complete each task. • Repeat task if subject cannot complete without support. • If completed without support proceed to next stance position. • It is unnecessary to perform Tandem position if patient fails Parallel position. • Instruct the patient to stand upright but relaxed with arms at sides and head up. • When standing with feet together in parallel, the big toes and heels should touch each other for duration of test. • When standing in tandem feet must be in a straight line without space between heel and toe. | |
| 0 | Normal: able to stand in all positions for 10 sec (without sway) |
| 1 | Mildly impaired: able to stand in normal and parallel positions 10 sec, but with sway and/or requires support in tandem stance |
| 2 | Moderately impaired: able to stand 10 sec in natural position, but cannot stand in parallel stance without support |
| 3 | Severely impaired: able to stand 10 sec in natural position, but requires intermittent support |
| 4 | Unable to stand in natural position 10 sec without constant support |
|  |  |
| Sitting | |
| Description: Item should be administered on an examination table. Back of legs may not be touching the table. Distance between calf and table should be about 1 inch. This item should be performed using the provided countdown timer set to 10 seconds. Ask the patient to sit with legs comfortably together. Knees do not need to be touching. Feet should not touch floor. Only sway of the trunk is rated (i.e., movement of limbs to maintain stability is not scored as truncal movement). It may be difficult to distinguish whether arm/head movements are due to arm/head tremor or due to sway of the trunk. In such instances, it is useful to concentrate on movement of the shoulders. Contrast between a score of 1 and 2 is based on whether the abnormal sway is continuous (i.e., present over the entire 10 seconds). If the sway is not present over the entire 10 seconds the score is a 1. | |
| 0 | Normal: no difficulties sitting 10 sec without sway or support |
| 1 | Mild difficulties: intermittent sway, but able to sit 10 sec without support |
| 2 | Moderate difficulties: constant sway, but able to sit 10 sec without support |
| 3 | Severely impaired: only able to sit for 10 sec with intermittent support |
| 4 | Unable to sit for 10 sec without continuous support |
| Speech | |
| Description: Score is based on conversational speech (e.g., discussion of recent events, activities, or other areas of interest). Scoring is determined by the rater’s ability to understand the patient, using the criteria listed below. Avoid using language test items or discussing the patient’s speech difficulties. | |
| 0 | Normal |
| 1 | Mildly impaired speech: easy to understand all words |
| 2 | Moderately impaired speech: occasional words difficult to understand |
| 3 | Severely impaired speech: with many words difficult to understand |
| 4 | Speech unintelligible or vast majority of words difficult to understand |

## Mapping of scores from SARA to f-SARA*

| SARA | f-SARA |
| --- | --- |
| GAIT | |
| 0, 0.5, 1 | 0 |
| 1.5 | 0.5 |
| 2, 2.5, 3 | 1 |
| 3.5 | 1.5 |
| 4, 4.5, 5 | 2 |
| 5.5 | 2.5 |
| 6 | 3 |
| 6.5 | 3.5 |
| 7, 7.5, 8 | 4 |
| STANCE | |
| 0, 0.5, 1 | 0 |
| 1.5 | 0.5 |
| 2 | 1 |
| 2.5 | 1.5 |
| 3 | 2 |
| 3.5 | 2.5 |
| 4 | 3 |
| 4.5 | 3.5 |
| 5, 5.5, 6 | 4 |
| SITTING | |
| 0 | 0 |
| 1 | 1 |
| 2 | 2 |
| 3 | 3 |
| 4 | 4 |
| SPEECH | |
| 0, 0.5, 1 | 0 |
| 1.5 | 0.5 |
| 2 | 1 |
| 2.5 | 1.5 |
| 3 | 2 |
| 3.5 | 2.5 |
| 4 | 3 |
| 4.5 | 3.5 |
| 5, 5.5, 6 | 4 |

* While 0.5 point steps are not part of the standard SARA administration for the SARA items in this table, half score values were observed in the natural history data for these items

## Clinical Global Impression – Global Improvement Scale (CGI)

Rate total improvement whether or not, in your clinical judgment, it is due entirely to drug treatment.

Compared to his/her condition at baseline, how much has he/she changed?

- 0 = Not assessed
- 1 = Very much improved
- 2 = Much improved
- 3 = Minimally improved
- 4 = No change
- 5 = Minimally worse
- 6 = Much worse
- 7 = Very much worse

## FARS Functional Staging for Ataxia

Increment by 0.5 may be used if the status is about the middle between two stages.

**STAGE 0:** Normal.

**STAGE 1.0:** Minimal signs detected by physician during screening. Can run or jump without loss of balance. No disability.

**STAGE 2.0:** Symptoms present, recognized by patient, but still mild. Cannot run or jump without losing balance. The patient is physically capable of leading an independent life, but daily activities may be somewhat restricted. Minimal disability.

**STAGE 3.0:** Symptoms are overt and significant. Requires regular or periodic holding onto wall/furniture or use of a cane for stability and walking. Mild disability. (Note: many patients postpone obtaining a cane by avoiding open spaces and walking with the aid of walls/ people etc. These patients are grades as stage 3.0)

**STAGE 4.0:** Walking requires a walker, Canadian crutches or two canes. Or other aids such as walking dogs. Can perform several activities of daily living. Moderate disability.

**STAGE 5.0:** Confined but can navigate a wheelchair. Can perform some activities of daily living that do not require standing or walking. Severe disability.

**STAGE 6.0:** Confined to wheelchair or bed with total dependency for all activities of daily living.

Total disability.

## PLS Regression Details

### Data preparation

- Subjects with a baseline SARA gait score of 0 or 8 were removed prior to conducting analyses.
- When mapping SARA to mod-SARA, 1/2 measures were retained and not rounded to integers.
- For all models, datapoints between 0 and 24 months were retained.
- Subjects were retained only if they met the following two criteria for each model:
- Data available for all included variables.
- Data available at baseline and 12 OR 24 months.
- All variables were scaled from 0 to 1 prior to computing change from baseline measures (ensuring that “1” represents the worse state on each given scale, swapping direction if necessary).
- Scaling of the CGI measure:
- In CRC-SCA, reported as a mixture of a 3-pt and 7-pt scale, while EUROSCA, reported as a 3-pt scale only.
- In order to ensure consistency between the datasets, scaling and computation of CFB measures were conducted simultaneously with the 3-pt scale being nested within the 7-pt scale.
- The scaled CFB measures for CGI therefore fell in [-1, -2/3, -1/3, 0, 1/3, 2/3, 1].

### Analysis Methods

- The dependent variable was defined continuous visit time variable (as opposed to categorized visit dates).
  - In CRC-SCA where there was more than a 6-month discrepancy between continuous and categorical time, continuous time was substituted with the categorical time measure.
- Items consisted of scaled (0 to 1) change from baseline (CFB) measures of select chosen variables for each model.
- The Variable Importance of Projection (VIP) was used for selecting the final set of items. VIP statistically summarizes the contribution each variable makes to the model. VIP cutoff was set to 0.5 or greater.
- The mean-to-standard deviation ratio (MSDR) was used to assess model performance:
  - MSDR from individual items at 12 months were compared to the overall composite score MSDR.
  - Overall composite score MSDRs were compared between models.
- PLS regressions were run using the *pls* (v2.8-1) package in *R*, while VIP scores were calculated using the *plsVarSel* (v0.9.9) package.
- An iterative process was used when fitting the PLS regressions:
  - An overall model was fit and each item with a negative weight was removed (indicates improvement on that item rather than progression).
  - Each remaining item with a VIP ≥ 0.5 was retained.
  - A model incorporating items with positive weights and VIP < 0.5 was fit.
- The % contribution from each item X was calculated as (weight_X/sum(all weights))*100.
- A final model was selected by considering both:
  - The overall MSDRs of models 2 and 3.
  - The % contribution of items with positive weights and VIP < 0.5.

If model 3 had a larger MSDR compared to 2 and the new items had a % contribution ≥ 5%, model 3 was selected. Otherwise, model 2 was retained.
